# Non-adherence to disease-modifying antirheumatic drugs is associated with higher disease activity in early arthritis patients in the first year of the disease

**DOI:** 10.1186/s13075-015-0801-4

**Published:** 2015-10-08

**Authors:** Annelieke Pasma, Charlotte V. Schenk, Reinier Timman, Jan J. V. Busschbach, Bart J.F. van den Bemt, Esmeralda Molenaar, Willemijn H. van der Laan, Saskia Schrauwen, Adriaan van’t Spijker, Johanna M. W. Hazes

**Affiliations:** Department of Rheumatology, Erasmus MC, University Medical Center Rotterdam, ’s-Gravendijkwal 230, 3015 CE Rotterdam, The Netherlands; Department of Psychiatry, section Medical Psychology and Psychotherapy, Erasmus MC, University Medical Center Rotterdam, ’s-Gravendijkwal 230, 3015 CE, Rotterdam, The Netherlands; Department of Pharmacy, Sint Maartenskliniek, Hengstdal 3, 6574 NA Nijmegen, The Netherlands; Department of Rheumatology, Groene Hart Hospital, Bleulandweg 10, 2803 HH Gouda, The Netherlands; Department of Rheumatology, Sint Maartenskliniek, Hengstdal 3, 6574 NA Nijmegen, The Netherlands; Department of Rheumatology, Sint Franciscus Gasthuis, Kleiweg 500, 3045 PM Rotterdam, The Netherlands

## Abstract

**Introduction:**

Non-adherence to disease-modifying antirheumatic drugs (DMARDs) hampers the targets of rheumatoid arthritis (RA) treatment, obtaining low disease activity and decreasing radiological progression. This study investigates if, and to what extent, non-adherence to treatment would lead to a higher 28-joint count disease activity score (DAS28) in the first year after diagnosis.

**Methods:**

Adult patients from an ongoing cohort study on treatment adherence were selected if they fulfilled the EULAR/ACR2010 criteria for RA, and were to start with their first DMARDs. Clinical variables were assessed at baseline and every 3 months. Non-adherence was continuously electronically measured and was defined as the proportion of days with a negative difference between expected and observed openings of the medication container out of the 3-month period before DAS28 measurement. Generalized linear mixed models were used to investigate whether the DAS28 related to non-adherence. Covariates included were age, sex, baseline DAS28, rheumatoid factor positivity, anti-cyclic citrullinated peptide antibodies (ACPA) positivity, anxiety, depression, weeks of treatment, number of DMARDs used, education level, use of subcutaneous methotrexate and biological use.

**Results:**

One hundred and twenty patients met the inclusion criteria for RA. During the study period 17 patients became lost to follow-up. There was a decline in adherence over time for all DMARDs except for prednisone. Non-adherence is a predictor of disease activity in the first 6 months of therapy, adjusted for weeks of treatment, baseline DAS28, and baseline anxiety.

**Conclusions:**

Non-adherence relates to disease activity. Therefore, interventions towards enhancing adherence can improve disease outcome.

## Introduction

Rheumatoid arthritis (RA) is a chronic autoimmune disease, which is characterized by joint inflammation with pain, swelling, damage and disability [1]. Early and adequate treatment with disease-modifying antirheumatic drugs (DMARDs) will prevent the disease from becoming worse. According to the guidelines, rheumatologists should strive for remission, or at least low disease activity within 3 months, in order to obtain the best functional and radiological outcomes [2, 3].

Adherence to DMARD therapy is important to reach the desired treatment outcome as stated in the guidelines, especially at the start of treatment. Following the guidelines for the treatment of RA, drug therapy should be adjusted at least every 3 months until the desired treatment target is reached [1, 3]. When patients are non-adherent to their drug therapy, it seems as if treatment fails, whereas in fact patients are not taking their medication. When treatment with synthetic DMARDs fails due to overlooked non-adherence, a step-up in therapy will be made. The first step-up is treatment with higher DMARD dosages or adding other synthetic DMARDs, and adds unnecessary risks to the treatment. When this step-up also fails, treatment with biological, and more expensive, DMARDs will be considered, adding even more costs and risks [3]. When prognostic unfavorable factors are present, an even earlier switch to biological DMARDs can be made according to the guidelines [3].

At the individual level, large differences in treatment response, as measured with the 28-joint count disease activity score (DAS28), are observed [4]. It is unclear which factors attribute to these differences. Studies have shown that amongst many factors, part of them are explained by age, sex, baseline DAS28 score, presence of rheumatoid factor (RF) or anti-cyclic citrullinated peptide antibodies (ACPA), type of treatment given, anxiety, coping with pain and locus of control (the extent to which patients believe they can control the pain) (Xiong H, Kuijper TM, de Jong PHP, Weel AEAM, Gerards AH, van Zeben J, et al.: Higher levels of baseline anxiety is a predictor of disease activity at three months in early arthritis patients initiating therapy with DMARDs, submitted) [5–10]. Non-adherence would also be a logical contributor to individual differences in DAS28.

Early and adequate treatment of RA will prevent the disease from becoming worse, and therefore adherence to the treatment is important for the management of RA. The consequences of non-adherence will not only affect the patient’s disease activity, but also the rheumatologist’s treatment decisions [11–13], which may lead to higher health care costs. Adherence in this early phase of disease has not been explored before. Furthermore, the extent to which non-adherence contributes to higher DAS28 in the first year of treatment is not yet determined. This study investigates if, and to what extent, non-adherence to DMARDs would lead to higher DAS28 scores in the first year after diagnosis.

## Methods

### Patients

A sample of 300 patients was consecutively recruited in 11 regional hospitals in the southwest of the Netherlands from January 2012 to July 2014 for a cohort study on DMARD adherence. Patients who were willing to participate were followed up for 1 year. Patients were included if they were at least 18 years old, were started on one or more DMARDs for RA for the first time, and were able to sufficiently read and understand the Dutch language. For the present analysis, we only selected those patients included before January 2014 and who were diagnosed with RA according to the European League Against Rheumatism (EULAR)/American College of Rheumatology (ACR) 2010 criteria for RA.

Participants in the study were, on a fixed time interval, seen by a research nurse or specialized rheumatology nurse after their regular rheumatologist consultation. Because the time interval in which the patient is seen by the rheumatologist differed per hospital, the time intervals vary. In the first year after diagnosis, RA patients are mostly seen every 3 months, but this time interval varied depending on the rheumatologist follow-up appointment.

The Erasmus MC medical research ethics committee gave their approval to perform the study. Each hospital’s board of directors gave their approval for participation in the study. All participants gave written informed consent for their participation and for retrieving relevant clinical data from their patient file.

### Primary outcome

Every 3 months, the DAS28 was measured by a trained rheumatology nurse. The score comprises four domains: swollen joint count (SJC), tender joint count (TJC), erythrocyte sedimentation rate (ESR) and a patient general health assessment using a visual analogue scale (VAS). For patients who dropped out of the study, but did not withdraw their consent, the DAS28 score was retrieved from the patient files.

### Clinical covariates

Clinical variables assessed at baseline included symptom duration before diagnosis, ACPA, RF, ESR (or C-reactive protein (CRP)) and joint involvement. ACPA and RF were combined for a RF/ACPA positivity score. Symptom duration was dichotomized in more or less than 6 weeks, according to the EULAR/ACR 2010 criteria for RA. The number of DMARDs used was counted and analyzed as a continuous measure. The use of either subcutaneous methotrexate (MTX) or biologicals was noted from the patient file and entered as a binary variable.

### Psychosocial covariates

Symptoms of anxiety and depression were measured at baseline with the Hospital Anxiety and Depression Scale (HADS) [14]. The questionnaire has two subscales: one for anxiety and one for depression. The scores range between 0 and 21, higher scores indicating more symptoms of anxiety or depression.

### Adherence measurement

Non-adherence was measured per DMARD using a ‘medication event monitoring system’ (MEMS) device, which consists of a medication vial and a MEMS cap. The MEMS uses a microprocessor in the medication container cap to record day and time of each vial opening. The data stored in the MEMS cap is transferred into a web-based data platform, which compiles hour-by-hour drug dosing histories, and in which medication regimen changes are noted. Indirect adherence measurement with MEMS is regarded as a gold standard, since it objectively measures a necessary behavioural step for adherence in ‘real time’ over a continuum. Disadvantages of using MEMS are the high price, the fact that it does not prove ingestion of medication and that it might be seen as an intervention, although this intervention effect is regarded as negligible [15]. Nursing and medical staff were blind to the adherence data throughout the study.

Extra openings of the MEMS cap were ignored, because these mostly do not represent medication intake, but openings by pharmacists. These would otherwise lead to an overestimation of adherence.

When patients stopped using one or more DMARD on the rheumatologist’s advice, for example in case of laboratory abnormalities, this was noted as a non-monitored period, which means that this period was not assigned as a non-adherence event.

For each individual patient and per DMARD, we calculated per day if there was medication underuse. Underuse was defined as a negative difference between the amount of observed openings minus the amount of expected openings. For MTX, we calculated the underuse not per day, but per week, since this medicine only needs to be ingested weekly. For the 12-week period before each DAS28 measurement, we calculated the proportion of days of DMARD underuse. If a patient used multiple DMARDs in the 12-week period, the mean underuse proportion was calculated. Adherence was also dichotomized using a non-adherence proportion above 0.2 (80 % or less adherence) and using a non-adherence proportion above 0.1 (90 % or less adherence).

When a patient used subcutaneous MTX, the patient was asked to put their folic acid in the MEMS container. The openings of the medication cap to take folic acid would then represent the use of subcutaneous MTX. Adherence to biologicals could not be measured. Patients that used biologicals also used other synthetic DMARDs to which adherence could be measured.

### Statistical analysis

Characteristics of the study population and non-adherence per DMARD were described with means, standard deviations, medians, interquartile ranges and percentages as appropriate. Four regression models were run with DAS28 as dependent continuous outcome; at T1, over the period T1 to T2 (two time points), the period T1 to T3 (three time points), and the period T1 to T4 (four time points) respectively.

First, univariate linear regression was performed for the T1 model to identify eligible predictors for the DAS28 score at T1. Predictors entered in the univariate regression were: standardized age, sex, baseline DAS28, RF/ACPA positivity, baseline anxiety, baseline depression, number of weeks using DMARDs, education level (low, medium or high), non-adherence, number of DMARDs used, use of subcutaneous MTX and biological use. Non-adherence and covariates with a *p* value lower than 0.2 were entered in the multivariate model.

For the influence of non-adherence on DAS28 over T1 to T2 (two time points), T1 to T3 (three time points) and T1 to T4 (four time points), multilevel regression models were performed with patients in the upper level and their repeated measures in the lower level. Variables that were taken into account in the models to predict DAS28 over time were: standardized age, sex, baseline DAS28, RF/ACPA positivity, baseline anxiety, baseline depression, number of weeks using DMARDs, education level (low, medium or high), non-adherence, number of DMARDs used, use of subcutaneous MTX and biological use. All possible predictors were entered in a univariate multilevel regression, taking into account the evolution of disease activity over time. Non-adherence and covariates with a *p* value lower than 0.2 were entered in the multilevel model. Because of potential collinearity between anxiety and depression, only one of these covariates will be included in the multivariate models.

If our study was a hypothesis-testing study, a Bonferroni correction should have been applied because of the number of possible covariates in the analysis. However, because of the explorative character of our study this requirement would be too strict, since then we would need a *p* value below 0.004 to reach statistical significance, and then no covariates would be left over.

A *p* value below 0.05 was considered statistically significant.

## Results

### Participants

**Fig. 1:**
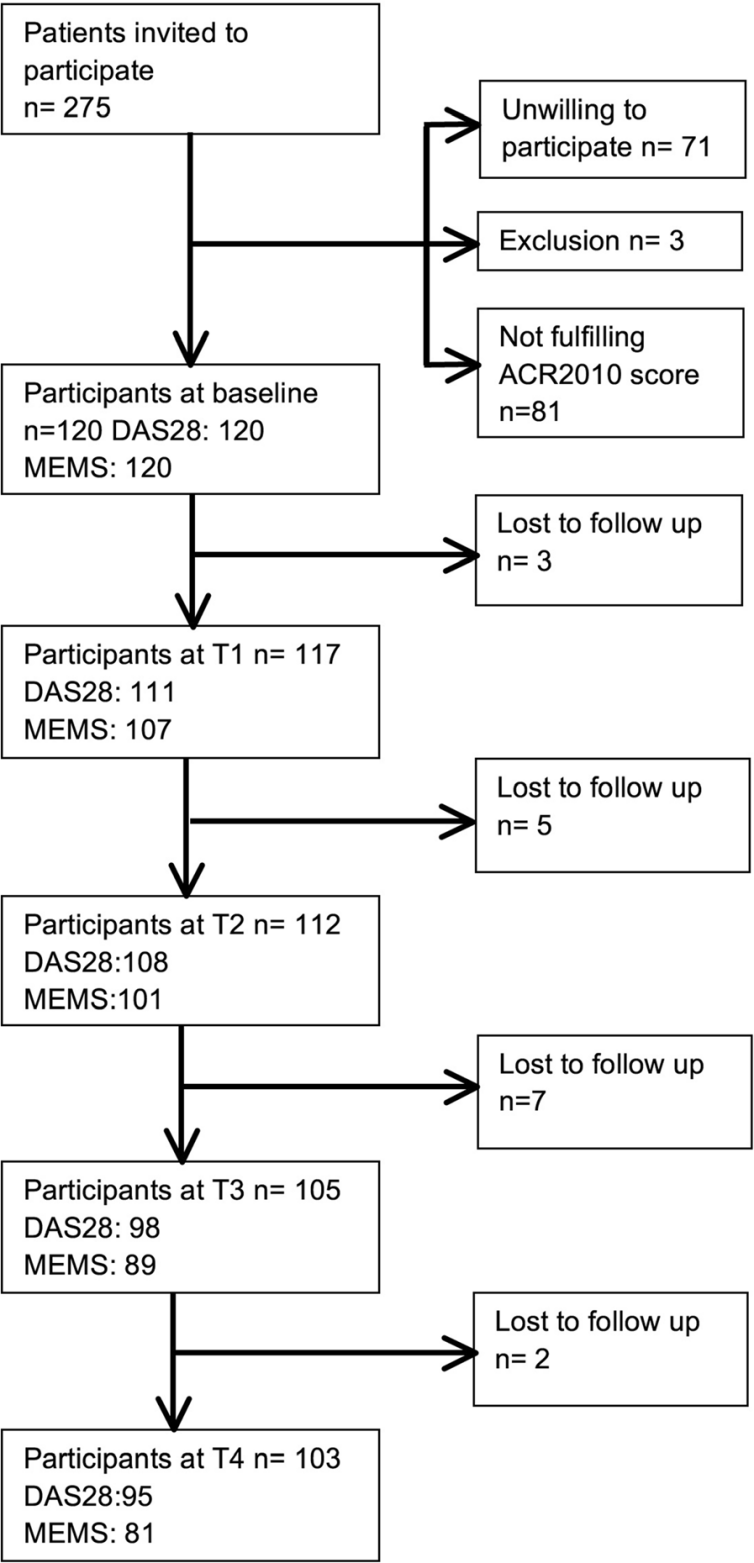
Flow chart of respondents

Before January 2014, 275 patients were asked to participate. Of those, 71 patients declined to participate and three were excluded. The EULAR/ACR 2010 criteria for RA were fulfilled by 120 of the 201 participants. During the study period, 17 patients became lost to follow-up (Fig. 1). Reasons for dropping out varied. Two patients stopped because of serious comorbidities, four patients did not show up at the study visits. Table 1 depicts the baseline variables. There are no significant differences between patients who became lost to follow-up and patients with complete follow-up.

**Table 1:** Baseline characteristics

		All patients	Patients with complete follow-up	Patients who became lost to follow-up
		*n* = 120	*n* = 103	*n* = 17
Age (years), mean (SD)		55.7 (13.2)	55.6 (13)	56 (15.3)
Sex, female, number (%)		80 (66.7)	71 (68.9)	9 (52.9)
TJC, median (IQR)		5 (2–11)	4 (2–11)	6 (3–10)
SJC, median (IQR)		3 (2–7)	4 (2–8)	3 (1–7)
ESR, mean (SD)		30.5 (23.3)	30.9 (24.1)	27.7 (17.9)
DAS28, mean (SD)		4.66 (1.3)	4.66 (1.29)	4.6 (1.4)
HAQ, median (IQR)		0.75 (0.38–1.13)	0.75 (0.38–1.14)	0.75 (0.25–1)
RF, % positive		93 (77.5)	79 (76.7)	14 (82.4)
ACPA, % positive		85 (70.8)	75 (72.8)	10 (58.8)
Symptom duration, >6 wk (%)		104 (86.7)	91 (89.2)	13 (81.3)
Number of DMARDs at baseline (%)	1	54 (45) 52	44 (42.7)	10 (58.8)
	2	39 (32.5)	34 (33)	5 (29.4)
	3	23 (19.2)	21 (20.4)	2 (11.8)
	4	4 (3.3)	4 (3.4)	–
Subcutaneous use of MTX during 1-year follow-up (%)		20 (16.7)	16 (15.5)	4 (23.5)
Use of biologicals during 1-year follow-up (%)		11 (9.2)	10 (9.7)	1 (5.9)
Education level	Low (%)	58 (50.4)	48 (48)	10 (66.7)
	Intermediate (%)	34 (29.6)	29 (29)	5 (33.3)
	High (%)	23 (20)	23 (23)	–
HADS depression, mean (SD)		4.5 (SD 2.7)	4.4 (2.7)	4.6 (2.9)
HADS anxiety, mean (SD)		5.7 (SD 4.4)	5.9 (4.5)	4.5 (4.1)

*SD* standard deviation, *TJC* tender joint count, *IQR* interquartile range, *SJC* swollen joint count, *ESR* erythrocyte sedimentation rate, *DAS28* 28-joint count disease activity score, *HAQ* health assessment questionnaire, *RF* rheumatoid factor, *ACPA* anti-cyclic citrullinated peptide antibodies, *DMARDs* disease-modifying antirheumatic drugs, *MTX* methotrexate, *HADS* Hospital Anxiety and Depression Scale

T1 (3 months) ranged from 5 to 20 weeks (mean 12 weeks), T2 (6 months) ranged from 16 to 33 weeks (mean 26 weeks), T3 (9 months) ranged from 20 to 49 weeks (mean 38 weeks) and T4 ranged from 41 to 68 weeks (mean 52 weeks). The distribution of weeks per time point was normal.

### Disease activity

**Fig. 2:**
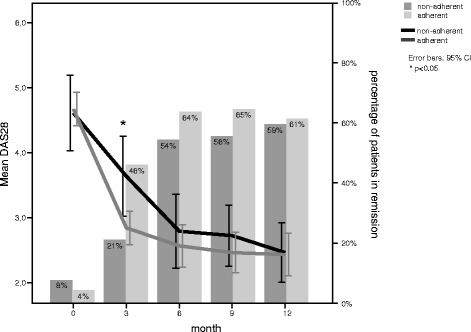
Mean disease activity and percentage of patients in remission for patients more or less than 10 % adherent

The mean DAS28 changed over time from 4.7 to 3.7 (3 months) to 2.7 (6 months), and 2.5 (9 and 12 months). For patients who became lost to follow-up during the cohort, the mean DAS28 improved more in the first 3 months of treatment (from 4.6 to 2.5), but worsened slightly after 9 months (from 2.5 to 3.0). Figure 2 depicts the course of the DAS28 over time for adherent (underuse proportion less than 0.1) and non-adherent (underuse proportion more than 0.1) patients. Non-adherent patients have a higher DAS28 (*p* = 0.01), especially at T2 (3 months). The proportion of patients achieving remission (DAS28 < 2.6) is at baseline 5, 41.4% at T2, 58.3 % at T3, 61.2 % at T4, and 57.9 % at T5. When we split the patients up into adherent and non-adherent, two times more adherent patients are in remission (Fig. 2) (*p* <0.05), especially at T2 (3 months of treatment).

### Non-adherence

**Fig. 3:**
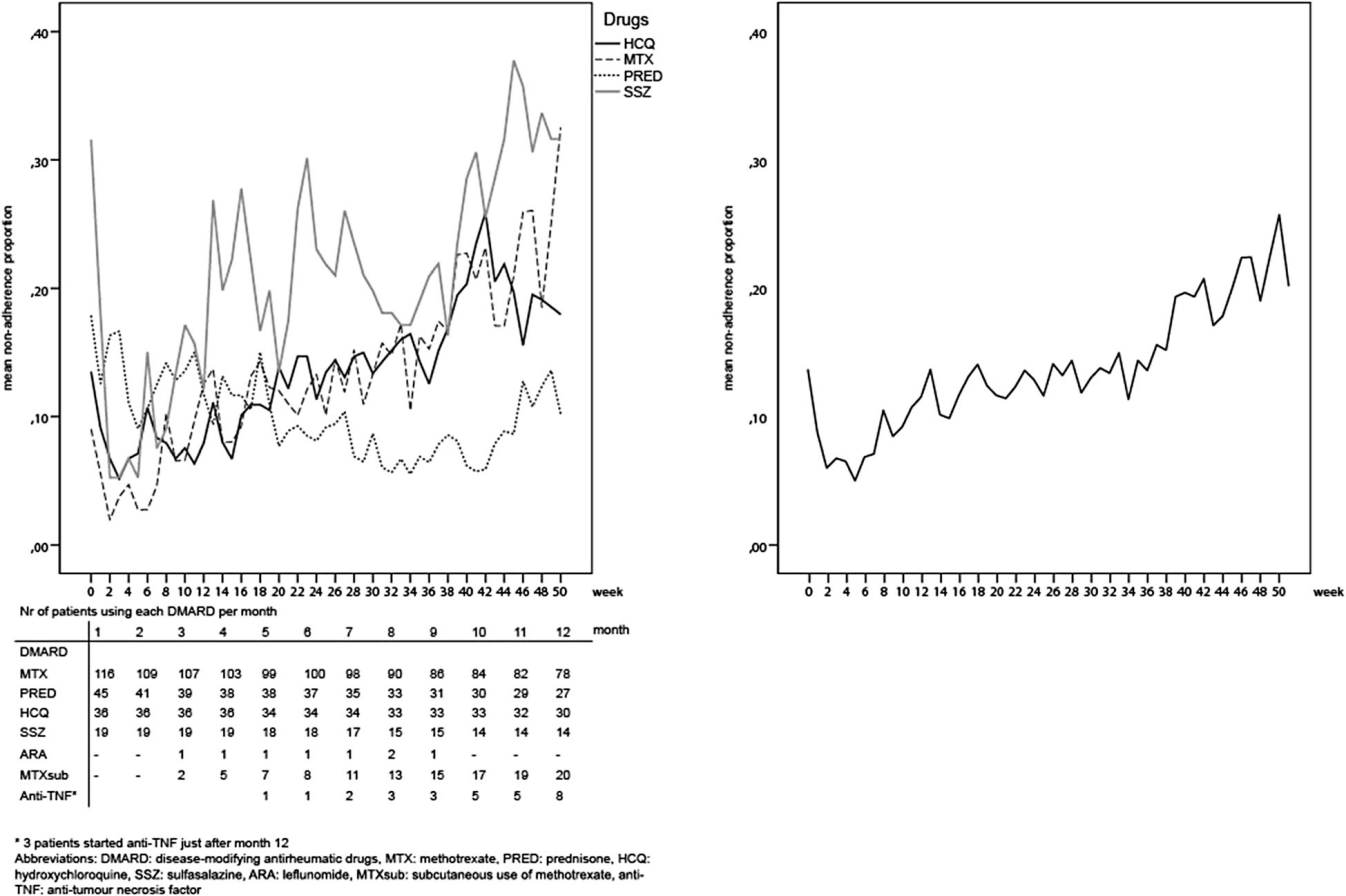
Non-adherence over the 1-year follow-up period

Most patients were started on monotherapy, of which MTX was prescribed the most often (43.3 %). A combination of MTX and prednisone bridging therapy with or without an additional DMARD was started for 32.5 % of the patients. Triple therapy following the O'Dell scheme was started for 13 patients (10.8 %). Mean non-adherence proportions increase over time (Fig. 3). Non-adherence proportions per DMARD also increased over time, except for prednisone (Fig. 3). Non-adherence proportions were highest for sulfasalazine. Methotrexate was the most used DMARD, but for this drug, the non-adherence proportion also increased to 0.3 after week 50. Using an 80 % adherence cutoff, for sulfasalazine the least patients were adherent, declining from 80 % (3 months) to 53.8 % (12 months). For MTX, adherence declined from 91.2 % (3 months) to 69.3 % (12 months). Over the study period, oral MTX was tapered the most. Two patients used leflunomide, which was not depicted in the graph.

#### T1: 3 months

Table 2 shows the univariate and multivariate regression analyses for each time period. In the multivariate regression model for DAS28 outcome at 3 months, non-adherence, weeks of treatment, baseline DAS28 and baseline anxiety were entered as predictors. The influence of non-adherence on disease activity is strongest in the first months of treatment. At T1, being non-adherent increases the DAS28 the most with 1.14 (95 % CI −0.07, 2.42), but is not significant with a *p* value of 0.08.

**Table 2:** Univariate and multivariate generalized linear mixed model for repeated data: determinants for DAS28 over the first year for rheumatoid arthritis patients over a 3-, 6-, 9- and 12-month period

	T1:				T1-2:				T1-2-3:				T1-2-3-4:			
	3 months				3–6 months				3-6-9 months				3–6–9–12 months			
	Univariate	*p* value	Multivariate	*p* value	Univariate	*p* value	Multivariate	*p* value	Univariate	*p* value	Multivariate	*p* value	Univariate	*p* value	Multivariate	*p* value
Intercept			2.02	<0.01			1.45	<0.01			1.57	<0.01			1.65	<0.01
Non-adherence	1.22	0.06	1.14	0.08	1.18	0.01	1.04	0.03	0.71	0.07	0.52	0.18	0.28	0.40	0.19	0.57
Weeks on DMARDs	−0.10	0.25	−0.08	0.10	−0.02	0.02	−0.02	<0.01	−0.02	<0.01	−0.02	<0.01	−0.01	<0.01	−0.01	<0.01
Baseline DAS28	0.36	<0.01	0.38	<0.01	0.35	<0.01	0.32	<0.01	0.32	<0.01	0.28	<0.01	0.30	<0.01	0.25	<0.01
Age (standardized)	0.01	0.17	0.001	0.92	0.02	0.07	0.01	0.29	0.02	0.03	0.01	0.11	0.02	0.03	0.01	0.11
Baseline anxiety	0.04	0.15	0.02	0.49	0.05	0.08	0.04	0.13	0.05	0.06	0.04	0.07	0.04	0.06	0.04	0.09
Education level	−0.17	0.63			−0.16	0.27			−0.16	0.23			−0.16	0.20		
Biological use	^#^				1.73	0.02	^#^		0.09	0.83			−0.09	0.78		
Subcutaneous MTX	0.38	0.51			0.43	0.23			0.32	0.24			0.16	0.51		
Gender	0.22	0.39			0.19	0.44			0.14	0.53			0.08	0.72		
Baseline depression	0.05	0.31			0.04	0.33			−0.03	0.43			0.02	0.62		
ACPA/RF	−0.11	0.40			−0.04	0.73			−0.04	0.74			−0.002	0.98		
Number of DMARDs	0.08	0.54			0.03	0.79			0.02	0.83			−0.02	0.84		
Symptom duration >6 weeks	−0.07	0.84			0.04	0.92			0.05	0.88			0.03	0.92		

*DAS28* 28-joint count disease activity score, *DMARDs* disease-modifying antirheumatic drugs, *MTX* methotrexate, *ACPA* anti-cyclic citrullinated peptide antibodies, *RF* rheumatoid factor

^#^No/only one patient received biologicals at this time point

#### T1-T2: 3 to 6 months

In the time period 3 to 6 months, non-adherence, weeks of treatment, baseline disease activity, standardized age and baseline anxiety were entered in the multivariate model. Non-adherence is a significant predictor of disease activity over time (independent of weeks of treatment, and baseline disease activity). Biological use was not taken into account in the multivariate model, since only 1 patient used biologicals at that time.

#### T1-T2-T3: 3 to 9 months

For the time period 3 to 9 months, non-adherence, weeks of treatment, baseline disease activity, standardized age, and baseline anxiety were entered in the multivariate model. Non-adherence was not a significant predictor of disease activity in this time period adjusted for the other variables.

#### T1-T2-T3-T4: 3 to 12 months

Over the whole first year of treatment, non-adherence and the same variables as in the 3- to 9-month model were entered in the multivariate model. Over this time period, non-adherence was also not a significant predictor of disease activity adjusted for the other variables.

### Other predictors

The number of weeks of treatment influences the DAS28 at all time periods. The longer the time on treatment, the lower the DAS28. The influence of time on treatment is the highest at T1 (3 months) and decreases when lengthening the time period.

The DAS28 score at the start of treatment is a predictor of the disease activity in the first year of treatment. The effect of the baseline DAS28 on the DAS28 decreases over time, but remains a significant predictor and lowers over time from 0.38 (T1) to 0.25 (T4).

Anxiety as measured with the HADS was multivariate not a significant predictor of disease activity.

## Discussion

The results of the present study indicate that non-adherence is a serious problem in the treatment of RA and that non-adherence corrected for other predictors hampers achieving remission in the first 6 months of treatment. Non-adherence increases over time for all DMARDs, except for prednisone. It was a strong predictor of higher disease activity and thus contributes to failure in obtaining remission. In addition, weeks on treatment and baseline disease activity influence the disease activity over time in the first year of treatment.

The effect of non-adherence disappeared after T2. A likely explanation for this effect is that if disease activity remained too high, a step-up in therapy was made following the treat-to-target principle, regardless of unknown underlying non-adherence behaviour [3]. Patients are probably more likely to adhere to the next step-up in treatment. An explanation for this is that they might need time to get used to taking medication or might be more adherent to more expensive and advanced therapy [16]. Unfortunately, we could not measure adherence to subcutaneous MTX and biological treatment, which was given to respectively 19.4 and 7.8 % of patients, so we could not confirm if this was in fact the case. The course of the DAS28 over the first year of treatment in our cohort is similar to that of other studies in which patients were treated according to the treat-to-target principle [17], which supports our explanation that patients were treated to target.

Time on treatment significantly influences the DAS28 at each time point. This is what we expect, because the more time the patient is on treatment, the lower the DAS28 is, especially in the early phase of treatment. The effect of time on treatment is highest after 3 months, and decreases during the course of the treatment. During the first months, time on treatment has larger effects than later in the course of the treatment, because the disease activity is then diminished.

Baseline disease activity is a significant predictor of the DAS28, regardless of the time period the patient has been treated. This is a known predictor of disease activity after 3 months [8], but no studies have been conducted on the influence of baseline disease activity on DAS28 after a longer period of time, except one study, which showed that baseline disease activity was a significant predictor of disease activity in established patients after a 2-year period [10]. There is a tendency for the effect of baseline disease activity becoming less over time, which is what we would expect when patients are treated to target.

Interestingly, non-adherence increased over time, except for prednisone. This is probably due to the immediate effect it has on the arthritis symptoms. For other DMARDs, it can take up to several weeks for an effect to be felt. It is logical that patients are more often non-adherent to drugs that have a delayed effect on the symptoms. For rheumatologists, it is important to be aware that patients are more often adherent to prednisone than to other DMARDs.

Although all patients in this study were diagnosed with RA according to the EULAR/ACR2010 criteria, there was high variability in treatment strategies used. From the data that we have, it is hard to determine if all patients were indeed treated according to the treat-to-target principles. Patients in this cohort might have been subjected to over- or undertreatment, which might increase or reduce the effects of non-adherence. Undertreatment occurs when the patient does not receive a step-up in treatment when needed according to the DAS28. Research has shown that rheumatologists’ non-adherence to the EULAR treatment guidelines in fact results in not obtaining remission [18]. In the case of undertreatment, being non-adherent will probably have larger effects on the DAS28 score, whereas in the case of overtreatment, the effect of non-adherence might be smaller. To overcome this possibility of confounding, we took in the regression analysis the number of DMARDs prescribed at each time point into account. When a patient has an increase in DAS28 and does not receive a step-up in treatment with additional DMARDs, this patient could be undertreated. In all the regression models, the number of DMARDs was not a significant predictor of disease activity. The effect of non-adherence still remained.

The outcomes of this study might have been subjected to the ‘adherer effect’ [19]. Patients who adhere to the rheumatologists’ prescription have better disease outcomes, regardless of the underlying treatment. This theory is based on the finding that behaviours of adherent people are different from the behaviours of non-adherent people. Adherent people have better global health outcomes, since they have more healthy lifestyles, do not engage in risky behaviours and are more adherent to non-pharmacologic prescriptions [10, 20, 21]. If we take this limitation into account, we can conclude that we are dealing with a rather adherent cohort. If more non-adherent patients had been in the study, there would have been more variation in adherence and maybe a stronger effect of non-adherence on disease outcome.

A limitation of this study is that the effect of patient education on adherence is unknown. Literature suggests that poor education about the disease and its treatment may have limited and short-term effects on non-adherence [22]. In our study, all patients received at least one education session from the specialized rheumatology nurse, unless they were unwilling to receive such education. However, the education given needs to be congruent with the patients’ lay beliefs [22]. We do not know whether this was the case. It may have been better if patient education was standardized over the participating hospitals and measured in this cohort. Another limitation is that there are a few missing DAS28 observations, which we chose not to impute. Because of the exploratory character of this study no multiple testing correction is applied. This might have caused arbitrary findings. However, if we had corrected for multiple testing our selection criteria for the multivariable model would have been too stringent. A strength of our study is that we measured adherence to DMARDs with the most accurate method we have up to now: electronic monitoring. Monitoring with MEMS might be seen as an intervention, but this effect is regarded as negligible [15]. Furthermore, patients can ‘cheat’ with MEMS, for example by opening and closing the pill box but not taking the prescribed medication. Nonetheless, electronic monitoring has been proven to be superior to patient self-reports and pill count in measuring adherence [23, 24]. In this study, electronic monitoring offered the advantage of studying adherence over a continuum, allowing selection of specific adherence period previous to DAS28 measurement. This would not have been possible with the use of questionnaires. Furthermore, using MEMS resulted in adherence data on the separate DMARDs, which would not have been possible if we had used conventional questionnaires.

During the follow-up time of the cohort, 17 patients became lost to follow-up. It could be that these patients are less adherent than the patients that completed the follow-up. We reviewed the patient files for information on the disease activity from the patients that became lost to follow-up. Strikingly, the disease activity from these patients after 3 months was 0.54 points lower than for the patients that stayed in the cohort, whereas the disease activity for these patients after 1 year was 0.58 points higher than for the patients who remained in the study. This finding suggests that these patients reached low disease activity relatively soon. Experiencing no or minimal symptom severity might trigger these patients to become less adherent, because they do not experience the need for taking their medication [16]. Non-adherence to their treatment might have caused higher disease activity in a later stage.

## Conclusions

This study showed that non-adherence is an important predictor of higher disease activity in the first 6 months of treatment. Since we know from the literature that it is important to reach remission as soon as possible to avoid permanent damage, the so-called window of opportunity, non-adherence needs extra attention especially in the first year of treatment. Rheumatologists should above all be aware that non-adherence is an important factor to take into account when treating the patient and evaluating DMARD efficacy and side effects. Shared decision-making is seen as an important overarching principle of care and was added to the European League Against Rheumatism (EULAR) recommendations for the management of rheumatoid arthritis in 2010 [1]. Shared decision-making is indeed a way in which the rheumatologist can improve patient adherence [25–27]. In daily practice, the rheumatologist should build towards an open and trustworthy relationship with the patient, in which non-adherence can be openly discussed. When the rheumatologist has a trusting relation with the patient, the rheumatologist will be able to know if non-adherence is hampering the treatment goal.

## Abbreviations

ACPA: anti-cyclic citrullinated peptide antibodies
CRP: C-reactive protein
DAS28: 28-joint count disease activity score
DMARDs: disease-modifying antirheumatic drugs
ESR: erythrocyte sedimentation rate
HADS: Hospital Anxiety and Depression Scale
MEMS: medication event monitoring system
MTX: methotrexate
RA: rheumatoid arthritis
RF: rheumatoid factor
SJC: swollen joint count
TJC: tender joint count
VAS: visual analogue scale
